# Juvenile Polyposis Syndrome in a Young Girl from Northern Tanzania

**DOI:** 10.1155/2020/1536090

**Published:** 2020-03-03

**Authors:** Jay Lodhia, David Msuya, Abdallah Msemo, Huda Akrabi, Patrick Amsi, Deborah Mchaile, Kondo Chilonga

**Affiliations:** ^1^Department of General Surgery, Kilimanjaro Christian Medical Center, PO Box, 3010 Moshi, Tanzania; ^2^Kilimanjaro Christian Medical University College, PO Box 2240, Moshi, Tanzania; ^3^Department of Pediatrics, Kilimanjaro Christian Medical Center, PO Box 3010, Moshi, Tanzania; ^4^Department of Internal Medicine, Kilimanjaro Christian Medical Center, PO Box 3010, Moshi, Tanzania; ^5^Department of Pathology, Kilimanjaro Christian Medical Center, PO Box 3010, Moshi, Tanzania

## Abstract

*Introduction*. Juvenile polyposis syndrome is a rare autosomal dominant disorder in children characterized by multiple polyps in the gastrointestinal tract. A variety of clinical features manifest, including prolapse of a polyp or entire rectum, gastrointestinal bleeding, anaemia, and intussusception. This condition if left unmanaged promptly leads to fatal complications including the development of cancer of the bowel. *Case Presentation*. A 13-year-old girl with a history of mass protrusion per anus associated with bloody diarrhea. Colonoscopy showed multiple polyps in her large bowel. She underwent total colectomy with ileorectal anastomosis and did clinically well post surgery with no complications. *Conclusion*. Juvenile polyposis syndrome is an inherited condition with significant morbidity and a high risk of colon malignancy. It is important for early screening and diagnosis and hence management in its early stages as there are no specific standard guidelines for children.

## 1. Introduction

Juvenile polyposis syndrome is a relatively rare autosomal dominant condition where hamartomatous polyps are found in the entire gastrointestinal tract but usually predominantly in the colon [1, 2]. Those with this condition are at a high risk of developing bowel cancer; however, the polyps are benign and, if left untreated, can bleed and lead to anaemia and cause other complications like rectal prolapse, intussusception, diarrhea, and malnutrition [2]. The risk of developing cancer ranges from 9 to 50% [3].

## 2. Case Presentation

A 13-year-old girl was referred to our center with a main complaint of generalized body swelling and difficulty in breathing for three months. The swelling started with both lower extremities and gradually became generalized. The girl also reported of awareness of heart beats and headache. The mother reported the child to have had several admissions due to mass protrusion through the anus with on and off history of bloody diarrhea since June 2018. She is the first born in the family of four, and her other siblings are well. Her maternal aunt also was reported to have had a history of a mass protruding through the anus.

Upon admission, the girl had bilateral lower limb grade three pitting edema with anasarca, finger clubbing, tinge of jaundice on the sclera, tanner stage two, and marked conjunctival pallor. Abdomen was tender with hepatosplenomegaly of 4 cm below the costal margins and a loose anal sphincter tone on digital rectal examination. On the cardiac examination, heart sounds one and two were heard with a grade two diastolic murmur, nonradiating. Initial complete blood count revealed leukocytosis of 26.7 × 10^9^ and moderate anaemia of 8.7 g/dl (microcytic hypochromic in nature). Serology for Hepatitis B and C surface antigen, ASOT, and blood for culture and sensitivity were negative.

Abdominal ultrasound was done and revealed free fluid in the abdomen and right lung pleural effusion. Doppler ultrasound of the right leg showed valvular insufficiency and cellulitis. Echocardiogram concluded a pericardial effusion of 12 mm and mild mitral and tricuspid valve regurgitation. The child was prescribed vitamin K intramuscular injection, intravenous Ceftriaxone, Captopril tablets, and Furosemide tablets and given multiple blood transfusions of whole blood.

During her stay in the wards, she was reviewed by the gynecology team whereby sexual abuse was ruled out. The progress in the ward was good with a decrease in oedema, but after a few days, the patient developed a bullae lesion on the right leg which progressed into an ulcer. Doppler ultrasound was done which showed occlusive thrombus from the distal third of the superficial femoral vein, including the popliteal vein and extending into the posterior tibial vein, and concluded deep vein thrombosis and cellulitis, and there was no feature of bone involvement. She was kept on thrombolytics and managed accordingly.

**Figure 1:**
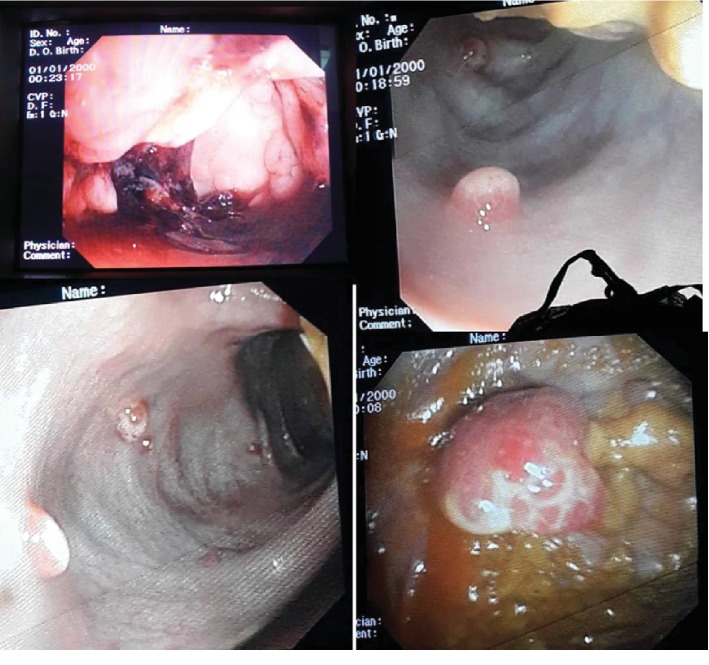
Colonoscopy showing polyps in the colon.

**Figure 2:**
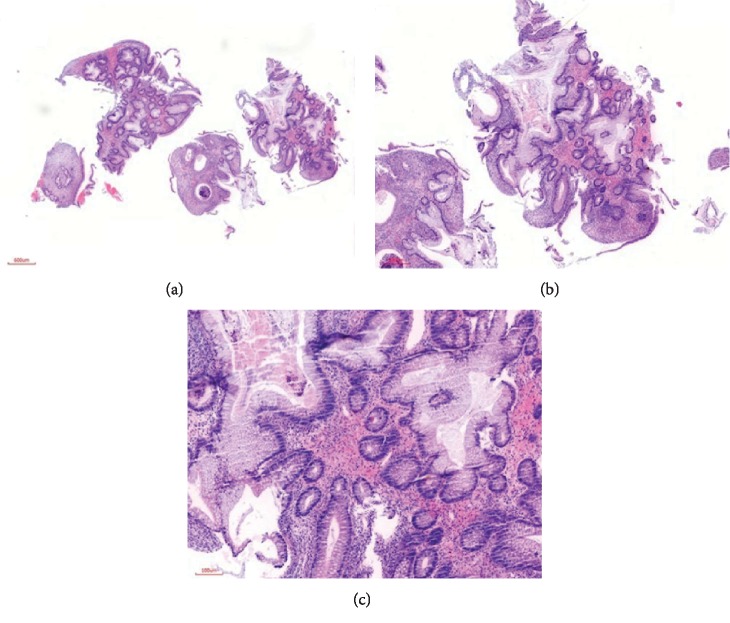
(a, b) H&E-stained sections of colon mucosal tissue showing a pattern of large dilated benign glands varying in size lying deep within the stroma (×40 and ×100 magnification, respectively). (c) H&E-stained section showing more details of the colon mucosal glands exhibiting benign mucinous columnar epithelial cells in a hemorrhagic stroma and surface epithelial erosion (×400 magnification).

**Figure 3:**
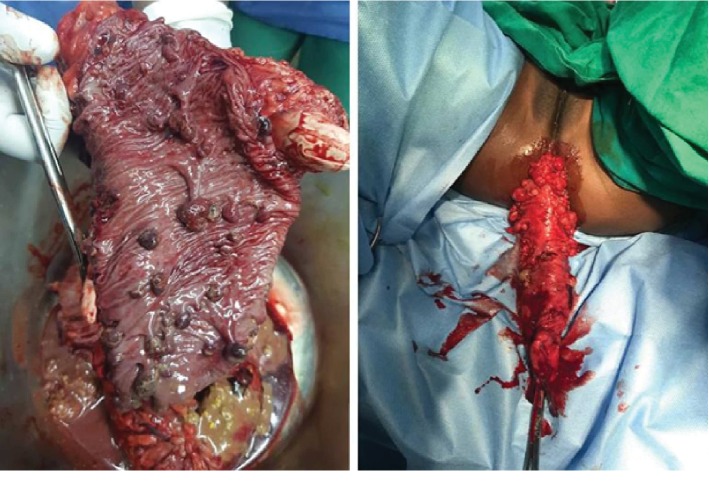
Photograph taken during surgery showing polyps in the colon.

Colonoscopy was done under general anesthesia which showed multiple polyps throughout her entire large bowel, highest concentration of polyps at the sigmoid (Figure 1). Biopsy was taken of the polyps during colonoscopy, and the histology confirmed juvenile polyposis syndrome (Figure 2). She was then optimized for surgery, where she underwent total colectomy followed by end-to-end ileorectal anastomosis, because of the extensive nature of the polyps throughout her colon hence not a suitable candidate for repeated colonoscopic polypectomy. Intraoperatively, the entire colon was found to have numerous polyps (Figure 3). Post operatively, she continued to do well in the general pediatric ward with no complications and was discharged two weeks after surgery. The child was reviewed two weeks post discharge and was doing well with a normal appetite, though reported an increased frequency of bowel movements but no diarrhea and no blood in stool.

## 3. Discussion

Juvenile polyposis syndrome is a rare disorder of the gastrointestinal tract described by the presence of hamartomatous polyps which are benign and have the tendency to become malignant, hence colon cancer [4]. The condition affects 1 in 100,000 to 1 in 160,000, and familial and sporadic autosomal dominant inheritance are found [5, 6]. This condition was first described in families in 1964, and currently, there are no specific molecular markers to differentiate sporadic from the syndrome-associated juvenile polyposis [5]. The genetic syndromes include juvenile polyposis syndrome, Cowden Syndrome (CS), and Bannayan Riley Ruvalcaba Syndrome (BRRS), and juvenile intestinal polyps are present in all [5]. Juvenile polyposis syndrome can further be clinically subdivided into Juvenile Polyposis of Infancy, Juvenile Polyposis Coli (colon involved only), and Generalized Juvenile Polyposis [2, 5].

Those with the condition have 50 to 200 polyps throughout the colon and sometimes in the stomach and small intestines. The polyps are few centimeters in size, smooth head with a stalk. Microscopically, they contain abundant lamina propria and tubules lined by a normal columnar epithelium [5]. In the review by Chow et al., the authors state that the risk of malignancy increases with age and there is no direct evidence to suggest the link between earlier onset of polyposis and a greater risk or earlier presentation of cancer. The lifetime risk of colon cancer is approximately 50% but very rare during childhood. A risk to other malignancies is not proven in juvenile polyposis [6]. Two particular genes (SMAD4 and BMPR1A) have been identified in juvenile polyposis syndrome that cause disruption of the growth factor TFG-*β* signal transduction pathway, and these genes are found in 50-60% of the patients [1, 3, 4, 7]. The PTEN gene has been identified in other syndrome-specific diseases like Cowden, Bannayan Riley Ruvalcaba, and Gorlin syndromes with developmental abnormalities and other tumors [5, 6, 8].

Management outline of the condition is genetic screening for the genes specified along with genetic counseling giving importance to family pedigree. Surveillance is equally recommended for bowel cancer by endoscopic means [3]. If few polyps are encountered, then they can be removed (polypectomy) endoscopically and taken for histopathology analysis to rule out any dysplastic changes [5]. For those with extensive polyps with or without complications or with malignant features in histology, surgical removal of the affected segment of the colon or total removal of the entire large bowel (total colectomy) is recommended; this was opted for the presented patient [4, 5, 7].

## 4. Conclusion

Juvenile polyposis syndrome is a rare condition featured by the presence of benign polyps in the gastrointestinal tract of pre- and school-aged children. They can present with anaemia from rectal bleeding, prolapsed of the rectum, or polyps per anus and if large enough can cause bowel obstruction. Genetic knowledge allows genetic diagnosis in about 40% with family pedigree screening.
